# Current therapeutic strategies for respiratory diseases using mesenchymal stem cells

**DOI:** 10.1002/mco2.74

**Published:** 2021-09-02

**Authors:** Ming‐yao Wang, Ting‐yue Zhou, Zhi‐dong Zhang, Hao‐yang Liu, Zhi‐yao Zheng, Hui‐qi Xie

**Affiliations:** ^1^ Laboratory of Stem Cell and Tissue Engineering Orthopedic Research Institute Med‐X Center for Materials State Key Laboratory of Biotherapy and Cancer Center West China Hospital Sichuan University and Collaborative Innovation Center of Biotherapy Chengdu China

**Keywords:** acute respiratory distress syndrome, asthma, chronic obstructive pulmonary disease, idiopathic pulmonary fibrosis, mesenchymal stem cells

## Abstract

Mesenchymal stromal/stem cells (MSCs) have a great potential to proliferate, undergo multi‐directional differentiation, and exert immunoregulatory effects. There is already much enthusiasm for their therapeutic potentials for respiratory inflammatory diseases. Although the mechanism of MSCs‐based therapy has been well explored, only a few articles have summarized the key advances in this field. We hereby provide a review over the latest progresses made on the MSCs‐based therapies for four types of inflammatory respiratory diseases, including idiopathic pulmonary fibrosis, acute respiratory distress syndrome, chronic obstructive pulmonary disease, and asthma, and the uncovery of their underlying mechanisms from the perspective of biological characteristics and functions. Furthermore, we have also discussed the advantages and disadvantages of the MSCs‐based therapies and prospects for their optimization.

## Graphical abstract

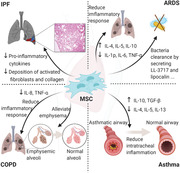

**Abbreviations**
ALI: acute lung injury
ARDS: acute respiratory distress syndrome
AT: adipose tissue
BM: bone marrow
BM‐MSCs: bone marrow mesenchymal stem cell
CCR5: CC‐chemokine receptor 5
CM: computational modeling
COPD: chronic obstructive pulmonary disease
COVID‐19: coronavirus disease 2019
CS: cigarette smoke
CXCR3: CXC‐chemokine receptor 3
EPA: eicosapentarnoic acid
ERK: extracellular signal‐regulated kinase
EVs: extracellular vesicles
FasL: Fas ligand
IA: intraarterial
IC: intracoronary
IGF: insulin‐like growth
IL: interleukin
IPF: idiopathic pulmonary fibrosis
iPSC: induced pluripotent stem cell
IV: intravenous
KC: keratinocyte‐derived chemokine
KGF: keratinocyte growth factor
MAPK: mitogen‐activated protein kinase
miRNA: microRNA
MMP: matrix metalloproteinase
MSCs: mesenchymal stem cells
MVs: microvesicles
NK: natural killer
OS: overlap syndrome
OVA: ovalbumin
SDF‐1: stromal cell‐derived factor‐1
TNF: tumor necrosis factor
UC‐MSCs: umbilical cord mesenchymal stem cells
UCTD: umbilical cord tissue source
VEGF: vascular endothelial growth factor

## 1 INTRODUCTION

The respiratory system plays a significant role in gas exchange, immune function, metabolism, and endocrine functions. However, owing to air pollution, smoking, population aging, and other factors, the incidence rate of respiratory diseases, especially respiratory inflammatory diseases, has increased, imposing a great financial burden on both national healthcare systems and citizens.

Respiratory inflammatory diseases include idiopathic pulmonary fibrosis (IPF), acute respiratory distress syndrome (ARDS), chronic obstructive pulmonary disease (COPD), and asthma,^1^, ^2^, ^3^, ^4^ which are usually characterized by inflammatory cell infiltration, cytokine release, epithelial cell damage, airway remodeling, and pulmonary tissue fibrosis.^5^, ^6^, ^7^ Dysregulation of the inflammatory response in the respiratory system, which is a significant cause of these diseases, is mediated by a complex intercellular interaction with concentration‐dependent regulation of various cytokines (especially inflammatory factors). With increasing cytokine concentrations, especially inflammatory cytokines, a devastating response can negatively affect the respiratory system.

Traditional drug intervention does not exert significant effects on the destructed airway and pulmonary epithelial cells or other pathological damages of the respiratory system caused by the inflammatory response. However, stem cells, especially mesenchymal stem cells (MSCs), have shown great potential in treatments, owing to their capacity to proliferate, undergo multidirectional differentiation, and exert immunoregulatory effects. Several preclinical studies have shown the significant therapeutic effects of systemic or endotracheal MSCs on a number of respiratory inflammation diseases. In some of these diseases, major breakthroughs have been made in the clinical application of stem cell therapy, thereby increasing our understanding of its therapeutic mechanisms and safety evaluation. Not only did Wei Jiang and Erin N Worthington^8^, ^9^ demonstrated the clinical safety of stem cell therapy, but also showed a statistically significant increase in the cure rate among patients receiving MSCs‐based therapy. As MSCs‐based therapy is being actively explored, the MSC characteristics and functions, which play key roles during the treatment, need to be summarized, and clarified in the context of therapeutic applications of MSCs.

A total of 397 articles, including 83 preclinical studies, and 28 clinical trials that included using MSCs to treatment respiratory immunological disorders, such as IPF, ARDS, COPD, and acute lung injury (ALI), were screened for this review. We summarized the properties and key functions of MSCs to clarify the corresponding treatment mechanism. Finally, we put forward the major challenges to the application of MSCs‐based therapy in respiratory inflammation diseases and further conclude appropriate improvement methods for its expansion.

## 2 THE CORE MECHANISMS UNDERLYING MSC THERAPY IN RESPIRATORY DISEASES

**FIGURE 1:**
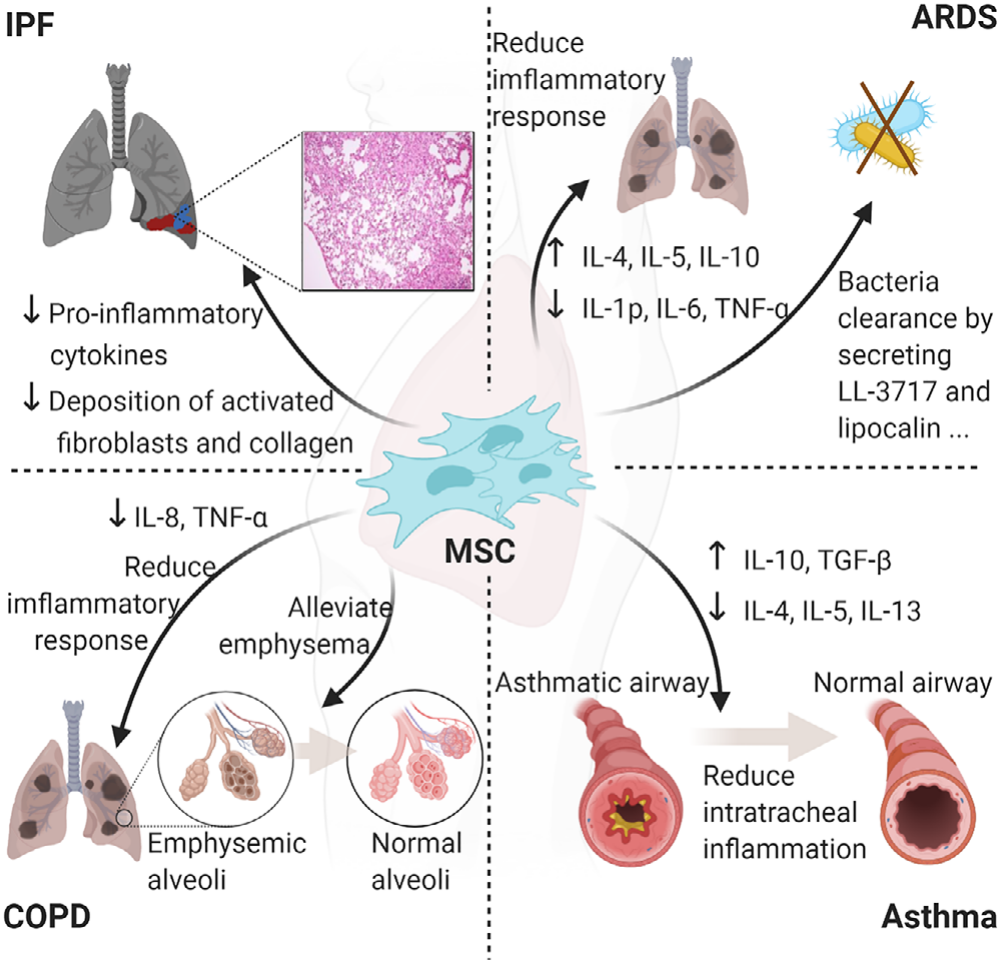
The dominant functions of MSCs in IPF, ARDS, COPD, and asthma

The properties of MSCs enable them to be used in the treatment of various diseases, including typical inflammatory diseases in the respiratory system. For example, immune compatibility allows MSC transplantation across histocompatibility barriers, which seldom causes immune response.^10^, ^11^, ^12^ Furthermore, like endogenous MSCs, exogenous imported MSCs can migrate to damaged tissues through the SDF‐1‐CXCR4 axis, in which stromal cell‐derived factor‐1 (SDF‐1) produced by the damaged lung tissue can bind to its receptor C‐X‐C motif chemokine receptor 4 (CXCR4) on the MSC to mediate the migration.^4^, ^5^ Moreover, MSCs can differentiate into type II alveolar epithelial (ATII)‐like cells through the activation of canonical and noncanonical Wnt pathways, thereby promoting the regeneration of damaged lung tissue.^13^, ^14^, ^15^ However, MSC differentiation potential has not been fully studied and, as MSCs can also differentiate into myofibroblasts and exacerbate pulmonary fibrosis under certain experimental conditions, the appropriate culture conditions should be found to allow MSCs to differentiate in the right direction.^16^, ^17^, ^18^ Additionally, the secretome of MSCs plays an important role in tissue regeneration and immunomodulation, which is believed to be the main mechanism by which MSCs can function in lung injury (Figure 1).

**FIGURE 2:**
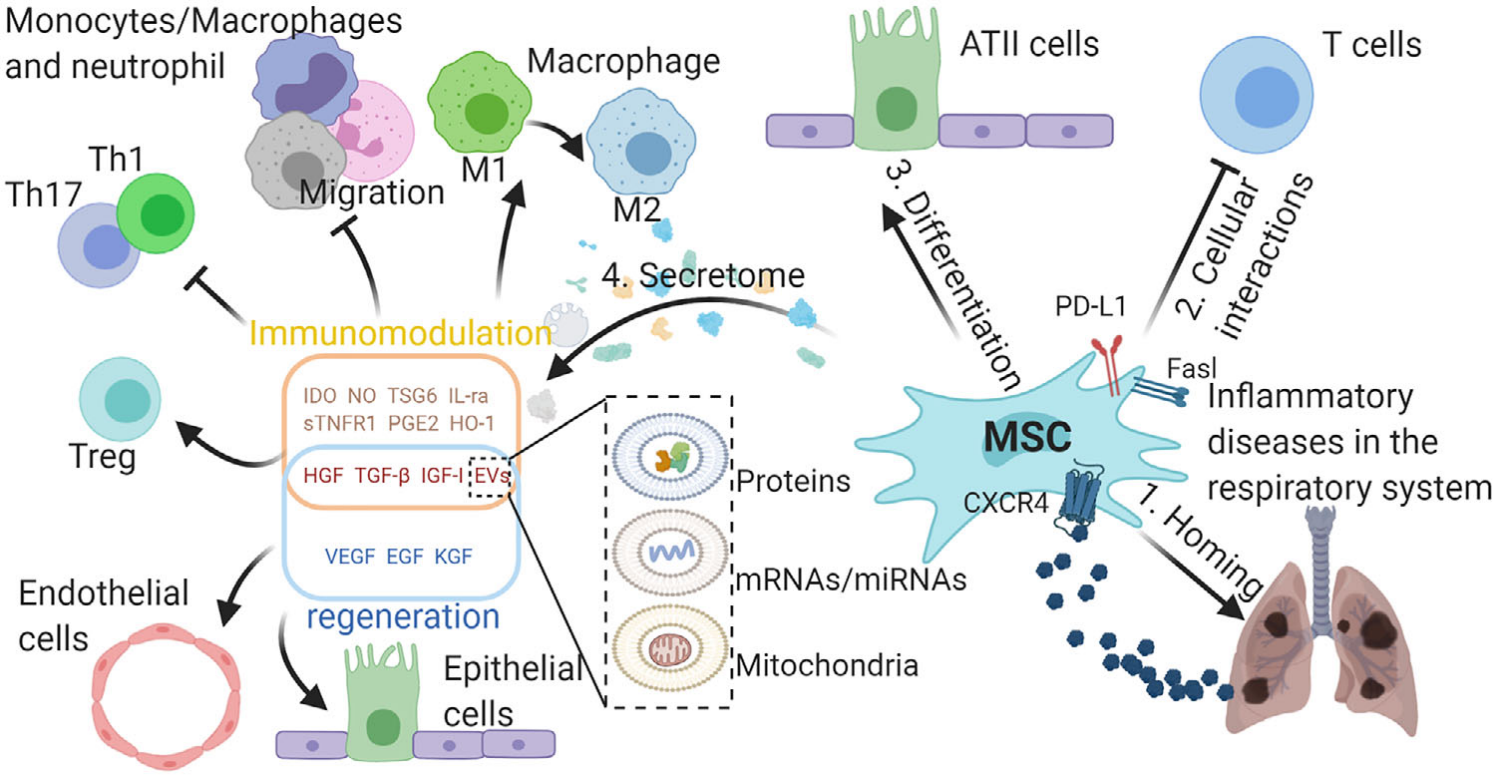
Properties of MSCs in typical inflammatory diseases in respiratory system. Exogenous imported MSCs can migrate to damaged tissues through the SDF‐1‐CXCR4 axis. MSCs can differentiate into ATII‐like cells and inhibit immune cells by cellular interactions and secret a variety of bioactive molecules which have immunomodulation abilities and can promote regeneration of the damaged tissue. The molecules associated with immunomodulation can shift macrophages from M1 to M2 phenotype, inhibit the migration of macrophages, neutrophil, and monocytes, inhibit the differentiation of TH1 and TH17 cells, and promote the formation of Treg cells. The growth factors can protect alveolar epithelial cells and pulmonary vascular endothelial cells from damage. MSCs can transport proteins, mRNAs/miRNAs, and mitochondria to other cells through EVs to exert its functions in immunomodulation and regeneration. The function of EVs is determined by its contents

Additionally, MSCs can regulate cell proliferation and control the secretion of various immune cells through cell–cell contact. In this section, we discuss in detail how MSCs perform roles in tissue regeneration and immunomodulation through the MSC secretome and cellular interactions between MSCs and immune cells (Figure 2).

### 2.1 MSC secretome

Currently, several studies have found that the paracrine function involving the secretion of soluble molecules and extracellular vesicles (EVs) by MSCs, rather than migration capability, and differentiation potential of MSCs, can promote the regeneration of damaged tissues and confer immunomodulatory capabilities, which may be the main mechanism by which MSCs play a beneficial role.

The soluble molecules produced by MSCs include growth and anti‐inflammatory factors, which mediate most MSC functions in inflammatory diseases of the respiratory system. MSCs can secrete a variety of growth factors to promote the repair of alveolar epithelial cells and pulmonary vascular endothelial cells. Under the stimulation of proinflammatory cytokines, such as tumor necrosis factor‐alpha (TNF‐α) and IL‐1β, the mRNA levels of growth factors produced by MSCs (including HGF, EGF, KGF, and VEGF) increased both in vivo^19^, ^20^ and in vitro.^21^, ^22^, ^23^ Moreover, the anti‐inflammatory factors produced by MSCs can interact with a variety of immune cell types in lung tissue^8^ and, after being stimulated by proinflammatory factors, MSCs produce various anti‐inflammatory factors, such as IDO and TSG6, to relieve inflammation. The exact mechanisms of action of these growth and anti‐inflammatory factors are shown in Table 1.

**TABLE 1:** The factors involved in the MSCs‐based therapy for inflammatory diseases in the respiratory system

Factors		Mechanism analysis	References
Growth factors	Epidermal growth factor (EGF)	Promote the proliferation of epithelial cells. Protect lung tissues from injuries by interacting with epithelial cells to make the epithelial cells produce secretory leukocyte protease inhibitor (SLPI), which is responsible for protecting local tissues against inflammation and acts by inhibiting proteases, such as elastase.	^20^, ^131^, ^208^
	Keratinocyte growth factor (KGF)	Prevent the damage of alveolar type epithelial(ATII) cells, cells treated with proinflammatory factors by activating the phosphoinositide 3‐kinase (PI3K) pathway of ATII cells to promote cell proliferation. Restore normal alveolar epithelial fluid transport. Facilitate repair of radiation‐induced DNA damage in alveolar epithelial cells.	^209^, ^210^, ^211^, ^212^
	Hepatocyte growth factor (HGF)	Exert an immunosuppressive effect by inducing monocytes to express IL‐10 and inhibiting T helper 1 (TH1) cells and dendritic cells (DCs) activity in many studies. Exert antibacterial effect in *E. coli*‐induced ALI mice model. Attenuate lung damage by promoting the proliferation of pulmonary vascular endothelial cells, inhibit their apoptosis, or reduce the expression of caveolin‐1 protein to maintain the integrity of the injured endothelial monolayer and restores lung vascular permeability.	^8^, ^18^, ^72^, ^74^, ^213^, ^214^
	Vascular endothelial growth factor (VEGF)	VEGF produced by MSCs or alveolar epithelial cells (under the stimulation of MSCs) can promote angiogenesis, which is essential for tissue repair because an adequate vascular network is required to supply blood and growth factors to injured tissues. Inhibit the apoptosis of pulmonary endothelial cells under pathological conditions to maintain the normal microcirculation.	^74^, ^76^, ^77^
Anti‐inflammatory factor	Indoleamine 2,3‐dioxygenas (IDO)	Relieve inflammation and significantly reduce lung neutrophil infiltration by catalyzing tryptophan, an important essential amino acid, into different metabolites, resulting in tryptophan depletion which can lead to T cell arrest by switching the metabolic pathway from glycolysis to oxidative phosphorylation. IDO metabolite KYNA can promote the expression level TSG6 in human MSCs by activating aryl hydrocarbon receptor (AhR) signaling in MSCs.	^39^, ^40^, ^75^
	Inducible NO synthase (iNOS)	Produce high concentration of NO and suppress the IL‐2 pathways by inhibiting phosphorylation of signal transducer and activator of transcription 5 (STAT5) thereby resulting in T‐cell proliferation and function inhibition in lung tissue.	^78^, ^79^
	TNF‐stimulated gene 6 (TSG6)	Reduce the proliferation of neutrophil and promote the formation of regulatory T (T_reg_) cells in injury lung tissue. Shift macrophages from a proinflammatory (M1) to an anti‐inflammatory (M2) phenotype in lung tissue by inhibiting the association of TLR2 with MYD88 and subsequently impairs NF‐κB‐dependent activation of proinflammatory gene transcription in macrophages.	^26^, ^36^, ^38^, ^80^, ^81^
	Transforming growth factor beta (TGF‐β)	Promote the formation of T_reg_ cells, which is responsible for the production of IL‐10. Suppress T_H_2‐driven allergic responses in the mice model of ragweed‐induced asthma. Play an antifibrosis role by activating IL‐6/STAT3 signaling, thereby increasing the expression level of antifibrotic chemokine IFN‐γ‐inducible protein 10 (IP‐10, also called CXCL10).	^83^, ^84^, ^85^
	Insulin‐like growth factor I (IGF‐I)	Decrease lung inflammation and protect lung tissue by promoting the polarization of macrophages from M1 phenotype to M2 phenotype.	^83^, ^164^
	Interleukin‐1 receptor antagonist (IL‐1ra)	Reduce the mRNA level of IL‐1α in lung, thereby blocking the proliferation of IL‐1α‐dependent T cells. Inhibit production of TNF‐α by activated macrophages.	^17^, ^42^
	Soluble tumor necrosis factor receptor 1 (sTNFR1)	Through the activation of NFκB pathway, MSCs may produce sTNFR1 to decrease a panel of inflammatory cytokines and inflammatory infiltration of macrophages and neutrophils in lung tissue.	^38^
	Prostaglandin E2 (PGE2)	Interacts with EP2 and EP4 receptors expressed on the surface of immune cells and exerts its anti‐inflammatory effects by promoting immune cells to express anti‐inflammatory cytokines such as IL‐10 and shift the macrophages into M2 phenotype.	^34^, ^35^
	Heme oxygenase‐1 (HO‐1)	Relieve lung inflammation by inhibiting the production of proinflammatory cytokines and protect human pulmonary microvascular endothelial cells from oxidative damage.	^86^, ^89^

EVs are naturally occurring, cell‐derived, membrane‐bound spherical structures that are shed or secreted from most cell types under various physiological and pathological conditions.^9^ The proteins, RNA (including mRNA and miRNA), and mitochondria contained in the exosomes and microvesicles (MVs) produced by MSCs may explain the beneficial effects of MSCs in typical inflammatory diseases of the respiratory system.

To some degree, the delivered components decide the function of EVs. First, it was indicated that exosomes may function at the protein level,^24^ as western blotting detected TSG6 (an anti‐inflammatory factor) in the exosomes secreted by MSCs. The exosomes isolated from TSG6 siRNA‐transfected MSCs lost their ability to reduce lung inflammation and protect tissues from damage in a mouse model of hyperoxia‐induced bronchopulmonary dysplasia.^24^ Second, Zhu et al. found that in a mouse model of endotoxin‐induced ALI, inflammation was alleviated, while KGF level was significantly elevated in lung tissue after administration of MSC MVs. After assessing the contents of MVs, there was not enough KGF protein to account for the level found in the lung tissue, indicating that MVs may function at the RNA level, perhaps by transferring KGF mRNA to the injured alveolar epithelium, which subsequently express the protein.^25^ Furthermore, microRNAs (miRNAs) are crucial components of EVs. The exosomes secreted by MSCs can prevent hypoxia‐induced pulmonary inflammation, and high expression levels of miRNA‐16 and miRNA‐21 were detected in these exosomes compared with those of the control group.^21^ These miRNAs have been shown to mediate the therapeutic effects of exosomes in different models.^22^, ^26^ Aliotta et al. performed miRNA microarray analysis on exosome‐based miRNAs and some miRNAs associated with anti‐inflammatory and antiproliferative effects, including miRs‐34a,‐122,‐124, and ‐127, were uniquely expressed or upregulated in exosomes secreted by MSCs.^23^

MSCs can also transport mitochondria to other cells through EVs.^27^, ^28^, ^29^ MSCs can modulate primary human macrophages through EV‐mediated transfer of functional mitochondria, which enhances macrophage oxidative phosphorylation. In a mouse model of LPS‐induced ALI, modulated macrophages expressed the M2 marker CD206 and reduced TNF‐α production.^29^ Mitochondria in EVs produced by MSCs can also be transferred into the alveolar epithelium, increasing ATP production in the epithelium and subsequently stabilizing the epithelium, and reducing lung inflammation.^27^

Through mass spectrometry and array analysis, more than 850 unique gene products and more than 150 miRNAs were identified in the cargo of MSC‐derived EVs.^30^ Some of them may mediate the protective effects of MSCs, but much remains unknown. Importantly, the content of EVs can change in response to cell activation, such as hypoxia, irradiation, injury, and cellular stress.^30^, ^31^ Thus, EVs produced under appropriate conditions may have better therapeutic value in cell‐free therapy.

The therapeutic effects of MSCs‐derived exosomes have been evaluated in several completed or ongoing clinical trials. Harrell et al. prepared an inhalation agent from exosome‐derived proteins, including sTNFRI, sTNFRII, IL‐1Ra, and sRAGE. Thirty adult COPD patients received this exosome‐derived protein inhalation agent and the treatment significantly improved their pulmonary status and quality of life.^32^ In an ongoing clinical phase I/II trial, 169 ARDS patients have been administered 2.0×10^8^, 8.0×10^8^, or 16.0×10^8^ exosome particles or normal saline per day for 7 consecutive days to test the efficacy of MSC exosomes (NCT04602104). Some other ongoing clinical trials are using exosomes in healthy volunteers to test their safety (NCT04313647). Sengupta et al. tested the efficacy of MSC exosomes in ARDS caused by SARS‐CoV‐2 infection. Reversal of hypoxia, immune reconstitution, and downregulation of cytokine storm was observed in patients who received a single intravenous dose of bone marrow (BM) MSCs‐derived exosomes, with no adverse effects attributable to the treatment.^33^ In a randomized double‐blind clinical trial conducted in Russia, COVID‐19 patients received exosomes (0.5×10^10^‐2.0×10^10^) or placebo (solution without exosomes). Although this trial was completed in November 2020, the results have not been published yet (NCT04491240).

### 2.2 Cells regulated by MSCs

Both innate and adaptive immune cells play important roles in the pathogenesis of inflammatory lung diseases. Immune cells, including eosinophils, macrophages, neutrophils, and T lymphocytes, among other immune cells, recruited by chemokines produced by the damaged lung tissue, may contribute to pulmonary inflammation. MSCs can interact with these inflammatory immune cells through their complex paracrine effects and surface molecules to attenuate lung inflammation.

Innate immune cells, including macrophages, neutrophils, dendritic cells, and natural killer cells, can be regulated by MSCs. For example, several factors released by MSCs, such as TSG6 and PGE2, can modulate macrophages, shifting them from a proinflammatory phenotype to an anti‐inflammatory phenotype.^26^, ^34^, ^35^, ^36^, ^37^ Macrophages can also engulf the EVs produced by MSCs, which contain functional mitochondria that enhance oxidative phosphorylation, thereby enhancing their anti‐inflammatory abilities.^29^ MSCs can also inhibit the infiltration of neutrophils in different ways; for example, indoleamine‐2, 3‐dioxygenase (IDO)‐dependent manner has been shown in several preclinical lung disease models.^38^, ^39^, ^40^

By modulating innate immune cells, MSCs can exert indirect regulatory effects on adaptive immune cells.^26^ For example, MSCs can switch the mature DCs into a suppressive immature phenotype and promote IL‐10‐positive pDC (plasmacytoid DC) differentiation, which results in the inhibition of effector T‐cells and the formation of T_reg_ cells.^8^, ^41^ MSCs can secrete a variety of anti‐inflammatory molecules, such as IDO, IL‐1ra, and PD‐L1, to inhibit effector T‐cells directly.^39^, ^40^, ^42^, ^43^, ^44^ Moreover, proinflammatory cytokine stimulated MSCs can produce ligands for CXC‐chemokine receptor 3 (CXCR3) and CC‐chemokine receptor 5 (CCR5), including CCL5, CXCL9, and CXCL11. These chemokines recruit T cells to the proximity of MSCs and, at the same time, MSCs secrete IDO/iNOS, inhibiting T cells in their vicinity.^26^, ^45^ By contrast, immunosuppressive factors secreted by MSCs can promote the formation of CD4^+^CD25^+^FOXP3^+^T_reg_ cells (e.g., transforming growth factor‐beta [TGF‐β]) and inhibit the differentiation of T_h_1 and T_h_17 cells (e.g., PGE2).

However, MSCs are not always immunosuppressive and do show proinflammatory properties under certain conditions. For example, MSCs can promote the infiltration of monocytes, macrophages, and neutrophils into tumors in a chemokine‐dependent manner.^46^ Studies have shown that an inflammatory environment with high levels of interferon gamma (IFNγ) and TNF is crucial for MSCs to exert anti‐inflammatory function. Low levels of IFNγ and TNF can endow MSCs with immunostimulatory potential.^45^ This characteristic of MSCs may explain why when MSCs are used to treat pulmonary fibrosis, they are only useful in the early stage of inflammation. However, when fibrosis has already occurred, MSCs cannot relieve inflammation because of the low inflammatory environment. This feature of MSCs emphasizes the necessity of using MSCs for treatment at the right stage. Additionally, for diseases with a low inflammatory environment,^47^ pretreatment of MSCs with proinflammatory factors before administration may improve their anti‐inflammatory effects.

## 3 SYSTEMATIC REVIEW OF MSCS‐BASED THERAPY FOR INFLAMMATION RESPIRATORY DISEASES

In this section, we have reviewed the application of MSCs for the treatment of respiratory inflammatory diseases, including IPF, ARDS, COPD, and asthma, with an emphasis on the significant breakthroughs made in the therapy and advances in our understanding of the mechanisms underlying MSC therapy (Tables 2, 3, 4, 5).

**TABLE 2:** The preclinic research of IPF over last decade

Year	Model	Animal	Cell source	Route	Outcome	References
2012	BLM induced	mice	ES	i.v.	Reduced inexpression of ECM and profibrotic genes.	^58^
2014	BLM induced	mice	BM‐MSC (STC1 plasmid)	i.t.	STC1 plasmid transfected to MSCs/STC1 inhalation as promising treatments for IPF	^58^
2015	BLM induced	mice	BM‐MSC	i.v.	Exhibited fibrosis (MMP‐2) activity, oxidative stress, apoptosis.	^151^
2016	BLM/orally induced	mice	BM‐MSC	i.v.	Immunomodulation and anti‐fibrotic effect at IPF early stage.	^88^
2018	BLM induced	mice	BM‐MSC with G‐CSF	i.v.	Promoted BM‐MSCs homing via upregulating CXCR4, increasing antifibrotic effects of BM‐MSCs.	^90^
2018	BLM induced	mice	BM‐MSC	i.t.	BM‐MSCs from IPF patients are senescent, inducing inflammation and senescence in the neighboring cells	^91^

**TABLE 3:** The preclinic research of ARDS over last decade

Year	Model	Animal	Cell source	Route	Outcome	References
2010	*E. col*i	mice	hBM‐MSC	i.t.	Improve IL‐37 level and decreased bacteremia and MIP‐2 level	^92^
2010	BLM induced	rat	BM‐MSC	i.v.	Suppressed inflammation and fibrosis, decreased IL‐6, IL‐10, IL‐1β, JE, TNF‐α, VEGF, TGF‐β	^216^
2010	CLP induced	mice	BM‐MSC	o.a.	Reduced cytokine levels in sepsis, prevented acute lung injury and organ dysfunction, down‐regulated inflammation (IL‐10, IL‐6) and swiftly up‐regulated phagocytosis and bacterial killing.	^118^
2010	LPS induced	mice	hBM‐MSC	i.v. or i.t.	ANG‐1 secretion prevented actin stress fiber formation and claudin 18 disorganization through NF‐κB suppression.	^216^
2011	LPS induced	mice	hBM‐MSC	i.t.	Anti‐inflammatory properties with secreting TSG‐6	^217^
2011	*E. coli* i.t.	mice	hUC‐MSC	i.t.	Attenuated E. coli‐induced ALI primarily by down‐modulating the inflammatory process and enhancing bacterial clearance.	^218^
2011	LPS induced	mice	hUC‐MSC	i.t.	Ameliorated ALI by diminishing CD4+CD25+ Foxp3+Treg and balancing anti‐ and pro‐inflammatory.	^219^
2012	CLP induced	mice	BM‐MSC	i.v.	Increased complementary activities in host cell metabolism and inflammatory response.	^220^
2012	*E. coli* i.t.	mice	BM‐MSC	i.t.	Enhanced survival and bacterial clearance in Gram‐negative pneumonia	^221^
2012	LPS induced	rat	hUC‐MSC	i.v.	Increased survival rate and reduced inflammation	^222^
2012	*P. aeruginosa* i.p.	mice	hBM‐MSC	i.v.	Increased survival in part by a monocyte‐dependent increase in bacterial phagocytosis	^223^
2012	hyperoxia	mice	BM‐MSC	i.p.	Produced soluble bioactive factors then regulated pathogenesis of inflammation and fibrosis	^224^
2013	VIL1	mice	BM‐MSC	i.t&i.v.	Enhanced recovery via a paracrine mechanism	^119^
2013	LPS induced	mice	BM‐MSC	i.v.	Improved lung function via inflammatory and remodeling	^225^
2013	LPS induced	rat	BM‐MSC	i.v.	Improved gas exchanges, alleviated the lung injury, and reduced mortality	^226^
2013	LPS induced	mice	hAT‐MSC (IL‐33/IL‐1 RL1)	i.v.	Reduced protein content, differential neutrophil counts, TNF‐α, IL‐6, and macrophage inflammatory proteins in bronchoalveolar lavage fluid	^227^
2014	*P. aeruginosa*	sheep	BM‐MSC	i.t.	Improved oxygenation, decreased pulmonary oedema	^228^
2014	Hyperoxia	rat	hUC‐MSC	i.t.	VEGF attenuated hypertoxic lung injuries	^229^
2014	CLP induced	rat	hBM‐MSC hUC‐MSC	i.v.	Increased circulating CD3+CD4+CD25+ Treg cells, enhanced suppressive function, and decrease serum levels of IL‐6 and TNF‐α	^230^
2014	*E. coli i.p*.	mice	AT‐MSC	Retro‐orbital	Ameliorated immune response with inflammatory cytokines decreasing and IL‐10 increasing, inhibited apoptosis and tissue damage	^231^
2014	LPS induced	mice	hBM‐MSC	i.v.	Regulated inflammatory response and inhibited lymphocyte proliferation	^232^
2014	CLP induced	mice	hUCB‐MSC (pcw poly(I:C))	i.v.	Poly(I:C) improved MSCs immunosuppressive abilities	^233^
2015	CLP induced	mice	hMens‐MSC (with antibiotics)	i.t.&i.p.	Enhanced survival by acting on multiples targets	^234^
2015	*E. coli* i.t.	rat	hBM‐MSC	i.v.	Decreased injury and reduced bacterial burden potentially via enhanced macrophage phagocytosis and increased alveolar LL‐37 concentrations	^235^
2015	CLP induced	mice	hBM‐MSC/murine BM‐MSC	i.v.	EPC‐EXP+MSC‐HUMAN yielded better lung function and reduced histologic damage	^236^
2015	VILI	rat	BM‐MSC	i.v.	Diminished injury and restoring lung structure, function	^237^
2015	*E. coli* i.t.	mice	hBM‐MSC/EV‐MSC	i.t&i.v.	MVs were as effective as the MSCs	^18^
2015	CLP induced	mice	Demal‐MSC	i.v.	Specific anti‐inflammatory effect on sepsis	^238^
2015	BLM induced	mice	BM‐MSC (pc hypoxia)	i.t.	Hypoxia‐preconditioned MSCs exerted better therapeutic effects and enhanced the survival rate, partially due to the upregulation of hepatocyte growth factor	^239^
2016	influenza A H5N1	mice	hBM‐MSC	i.v.	Prevented or reduced injury in vitro and in vivo	^127^
2016	CLP induced	rat	hWJ‐MSC	i.p.	Klotho protein expression was higher in WJ‐MSCs than in hAD‐MSCs	^112^
2016	*E. coli* i.t.	mice	hBM‐MSC/EV‐MSC	i.t&i.v.	Innate immune cells enhanced phagocytic activity	^240^
2016	*E. coli* i.t.	mice	hUC‐MSC	i.t.	BD2 secreted then ensued acute lung injury through anti‐inflammatory and antibacterial effects	^218^
2016	LPS induced	rat	BM‐MSC	i.v.	Restored the lung permeability and attenuated lung injury by maintaining VEGF level	^241^
2017	CLP induced	rat	hUC‐MSC	i.v.	Reduced rodent mortality after induced ARDS‐SS	^242^
2017	LPS induced	mice	AT‐MSC	Retro‐orbital	Modulated inflammatory response and oxidative damage	^243^
2017	LPS induced	mice	hMens‐MSC	i.v.	Upregulated PCNA, KGF and down‐regulated caspase‐3, then improved BEAS‐2B cells viability and inhibited LPS‐induced apoptosis	^244^
2017	LPS induced	mice	hUC‐MSC (with FTY720)	i.v.	Showed higher survival and attenuated lung injuries	^245^
2018	LPS induced	mice	Huc‐MSC (with FTY720)	i.v.	Mitigated LPS‐induced inflammatory lung injury	^246^
2018	LPS induced	rat	BM‐/AT‐/lung‐derived MSC	i.v.	BM‐MSCs and AT‐MSCs yielded greater effects than lung tissue MSCs	^247^
2018	LPS induced	rabbit	BM‐MSC	i.t.	Reduced inflammatory cells, pulmonary hemorrhage and edema infiltration	^248^
2019	HCI/VILI	mice	BM‐MSC	i.t&i.v.	Retained protective effects in small airway epithelial cells	^120^
2018	LPS induced	mice	BM‐MSC (oe IL‐10)	i.t.	IL‐10‐MSCs offered superior protection against LPS‐induced ALI	^249^
2018	LPS induced	mice	hAM‐MSC (oe Nrf2)	i.v.	Nrf2 overexpression reduced lung injury, fibrosis, and inflammation	^250^
2019	LPS induced	rat	Lung‐derived MSC	i.v.	Upregulated the balance of Tregs and Th17 cells	^251^
2019	LPS induced	rat	BM‐MSC (oe HO‐1)	i.v.	MSCs‐HO‐1 enhanced protective effect	^89^
2019	HCl/VILI/both	mice	BM‐MSC (oe HGF/IL‐10)	i.t.	Effects depending on micro‐environment at the time of administration	^120^
2019	*E. coli* i.t.	rabbit	hUC‐MSC (oe IL‐10)	i.v.	IL‐10 overexpression in UC‐MSCs enhanced their effects in E. coli pneumosepsis and increased macrophage function	^252^
2019	LPS induced	mice	BM‐MSC (kd Lats)	i.t.	Inhibited Hippo by Lats1 knockdown improved the protective effects against epithelial damage and the therapeutic potential	^253^
2019	LPS induced	mice	hBM‐MSC/EVs (pdw serum from injured mice)	i.v.	EPA‐preconditioned AT‐MSCs yielded further reductions in injury, resulting in greater improvement and higher survival rate	^254^
2019	CLP induced	mice	AT‐MSC (pdw EA)	i.v.	MSCs yielded greater overall improvement in ARDS in comparison to EVs	^255^
2019	LPS induced	mice	BM‐MSC (oe p130/E2F4)	i.t.	Over‐expressing p130 or E2F4 in BM‐MSCs could further improve the injured structure and cells function	^256^

**TABLE 4:** The preclinic research of COPD over last decade

Year	Model	Animal	Cell source	Route	Outcome	References
2010	papain induced	rat	BM‐MSC	i.v	Protected pulmonary emphysema by secretion of reparative growth factors	^77^
2011	cigarette smoke induced	rat	BMC vs. BM‐MSC	i.v.	BMC better induced AT2 cells and pulmonary vascular endothelial cells proliferation; BM‐MSC and BMC both alleviate emphysema	^200^
2013	cigarette smoke induced	rat	BM‐MSC	i.t.	Down‐regulated COX2 and E‐2 by p38 and ERK MAP kinase activity inhibition in macrophages	^74^
2014	elastase or cigarette smoke induced	mice	BM‐MSC, AT‐MSC, LT‐MSC	i.v. i.t.	Decreased keratinocyte‐derived chemokine and TGF‐β levels in all sources, i.v. with better cardiovascular function and phenotype change from M1 to M2.	^10^
2015	elastase or cigarette smoke induced	mice	hAT‐MSC	i.v.	AT‐MSC decreased mean linear intercept and reduced caspase‐3 activity	^257^
2016	elastase induced	mice	hBM‐MSC	i.v.	Reduced fibrosis, reduced inflammation	^258^
2016	cigarette smoke induced	mice	hBM‐MSC, UCB‐MSC	i.v.	Reduced inflammation, increased regeneration	^259^
2017	cigarette smoke induced	mice	hBM‐MSC/Ipsc	i.v.	Reduced inflammation	^260^
2018	elastase induced	mice	LT‐MSC	i.t.	Activated HGF/c‐Met system, by promoting survival and proliferation of alveolar epithelial cells	^261^
2018	elastase induced	mice	BM‐MSC	i.t.	Attenuated pulmonary arterial hypertension	^262^
2019	elastase induced	mice	hWJ	i.v.	Promoted regenerative effect, pathomechanism not investigated	^263^
2019	overlap syndrome (OS)	rat	BM‐MSC	i.v.	Inhibited emphysema progression by differentiating into endotheliocytes, suppressing the apoptosis of endotheliocytes and oxidative stress	^264^

**TABLE 5:** The preclinic research of Asthma over last decade

Year	Model	Animal	Cell source	Route	Outcome	References
2010	ragweed induced	mice	BM‐MSC	i.v.	BM MSC suppressed Th2‐driven allergic responses by TFG‐ β production	^84^
2012	ovalbumin induced	mice	h iPSC vs. BM‐MSC	i.v.	iPSC‐MSC owned same therapeutic effect as BM MSC	^265^
2015	house dust mite induced	mice	BM‐MSC	i.v.	Reduced eosinophilia, Th2 response and activated dendritic cells	^266^
2015	ovalbumin induced	mice	BM‐MSC	i.v.	Reduced lymphocytes and eosinophils attraction, suppressed lung dendritic cell maturation and Th2 responses	^267^
2016	ovalbumin induced	mice	BM‐MSC	i.v.	Reduced neutrophils and eosinophils recruitment, goblet cell hyperplasia and lung fibrosis	^268^
2016	house dust mite induced	mice	BM‐MSC	i.v.	Expressed high levels of COXZ (MSCs), IL‐10 and TGF‐β (M2 macrophages) and low level of IL‐6	^269^
2017	house dust mite induced	mice	BM‐MSC	i.t.	Reduced inflammation, remodeling, and improved lung functions	^135^
2017	ovalbumin induced	mice	hUCB‐MSC	i.v.	Reduced allergic inflammation which mediated by regulatory I cells	^270^
2018	ovalbumin induced	mice	BM‐MSC vs. AT‐MSC	i.t.	Therapeutic efficiency only after BM‐MSC treated	^136^
2018	ovalbumin induced	rat	hPD‐MSC	i.v.	Shifted from Notch‐1, ‐2 and jagged‐1 to Notch‐3, ‐4 and delta‐like ligand‐4 signaling	^271^
2018	ovalbumin induced	mice	mASC‐	i.t.	Alleviated airway inflammation, improved airway remodeling, and relieved airway hyper‐responsiveness with the restoration of Th1/Th2 cell balance	^16^
2019	ovalbumin induced	mice	BM‐MSC	i.v.	Simvastatin and BMSCs affected serum IgE, lung IL‐13 and TGF‐β levels more than BMSC. Simvastatin increased BMSCs migration into the lung tissue	^272^
2019	Th2‐mediated inflammation induced	rats	rBM‐MSC	i.t.	Ameliorated pathological changes presumably by targeting ICAM‐1 and VCAM‐1	^13^

### 3.1 Idiopathic pulmonary fibrosis

IPF is an unexplained and chronic progressive pulmonary fibrosis disease,^48^ predominantly occurring in middle‐aged and elderly men with long‐term smoking history.^49^ Moreover, its prevalence and mortality has been steadily yearly,^50^ and its etiology and clinical manifestations remain unclear.^51^ As a significant pathological feature, it causes diffuse alveolar inflammation, characterized by fibroblast proliferation and transformation in the lungs, extracellular matrix expansion, and extensive lung parenchyma refactor.^52^, ^53^ Pulmonary fibrosis is closely related to immune disorders. Inflammatory stimulation leads to the proliferation of inflammatory cells, releases a large number of cytokines, such as TNF‐α, platelet‐derived growth factor (PDGF), TGF‐β, insulin‐like growth factor 1 (IGF‐1), interleukin (IL)‐4, ET‐1, and connective tissue growth factor, and then stimulates fibroblast pathogenesis and collagen accumulation.^54^ As the mechanism is being gradually unraveled, the IPF model construction has constantly improved and the application scope of stem cell therapy in IPF has expanded.

Regarding preclinical research, the bleomycin‐induced fibrosis model is most commonly applied to evaluate the potential therapeutic effect of MSCs on IPF,^55^ showing MSC treatment to have a remarkable effect (Table 2). Intravenous (IV) injection of human BM MSCs is more effective than traditional drugs in improving bleomycin‐induced pulmonary fibrosis and the feasibility of combination therapy.^56^ Furthermore, administration of BM‐ or umbilical cord (UC)‐MSCs early in the initial inflammatory phase can have a protective effect, reducing the levels of proinflammatory cytokines and deposition of activated fibroblasts and collagen, while improving epithelial repair. Moreover, several well‐designed clinical trials of MSC treatment for IPF patients have been steadily carried out. The most relevant research projects focused on allogeneic BM‐MSCs,^57^ and placental‐derived MSCs^58^ have also been used in clinical trials. Only minor adverse events have been reported, and there is no evidence of worsening fibrosis.^58^ In addition, high‐dose MSCs are safe for IPF treatment and can delay disease progression to a certain extent.^59^

### 3.2 ARDS

ARDS is an acute progressive respiratory failure caused by changes in lung tissue structure owing to various factors. It can be caused by infectious factors, such as bacteria, viral infections, and noninfectious factors, such as inhalation of toxic gases and massive blood transfusions. Severe cases can further develop non‐ARDS. The mortality rate can be as high as 30−40%, and morbidity and mortality increase with age.^60^, ^61^ ALI can cause alveolar epithelium and vascular endothelial cell damage, as well as increased pulmonary vascular permeability. The main clinical manifestations are low oxygenation, low lung compliance, and high physiological dead space. Its pathogenesis is complicated and the currently recognized mechanism involves various causes leading to increased production of proinflammatory factors in the lungs, followed by massive release and activation of inflammatory factors drive the inflammation in the lungs out of control.^62^ Whether the inflammatory response subsides effectively or not is key in the treatment of ARDS patients. Current treatment mainly focuses on the regulation of inflammatory response by stimulating nuclear factor‐kappa B and inhibiting the glucocorticoid receptor‐mediated signal transduction cascade.

Regarding preclinical research, the experimental results obtained by different models have their own advantages and disadvantages, but all have shown the advantages of MSC therapy in ARDS treatment (Table 3). Methods used in these models mainly include treatment with lipopolysaccharide/cefpodoxmine proxetil (CLP)/hypoxia pretreatment and live virus infection, whereas in vivo models have been based on animals, including mice and rats (small animal‐based models), and sheep^63^ and pigs,^64^ among others(large animal‐based models). Although the underlying mechanism remains unclear, results have shown that MSCs can reduce inflammation significantly and restore lung functions.

To date, 13 clinical trials of MSC therapy in ARDS patients have been registered. Although all of them are in the early phase and are limited by small sample size, they still successfully assessed the safety of MSC administration and treatment efficiency in clinical outcomes, for example, respiratory and systemic parameters, inflammation, and hemodynamics. Several recent clinical studies have confirmed that MSC injection relieves ARDS symptoms in patients with COVID‐19.^65^, ^66^ However, as most experimental studies, these clinical investigations present substantial heterogeneity concerning inclusion and exclusion criteria, follow‐up length, MSC dose, source, route of administration, and treatment frequency.^67^

### 3.3 Chronic obstructive pulmonary disease

COPD is one of the most common chronic lung diseases in humans. Its high prevalence is one of the leading causes of morbidity and mortality worldwide. COPD is characterized by persistent respiratory symptoms and airflow obstruction, which is caused by chronic bronchitis and destruction of the lung parenchyma (emphysema). Changes in the function of immune cells infiltrating the lungs, oxidative stress, and imbalance in the activity of proteases and their inhibitors are the main causes of major pathological changes in COPD patients.^68^

For preclinical research on COPD, a COPD rat/mouse model induced by elastase or cigarette smoke (CS) is most commonly used. These studies have shown that MSCs can alleviate emphysema and inflammation symptoms and have a good therapeutic effect in experimental COPD (Table 4). MSCs from different sources can reduce the mean linear intercept, neutrophil infiltration, and cell apoptosis; increase elastic fiber content, reduce alveolar epithelial and endothelial cell damage, and decrease keratinocyte‐derived chemokine, that is, a mouse analog of IL‐8, and TGF‐β levels in lung tissue.^10^, ^69^

In terms of alleviating emphysema, the role of MSCs has been mainly reflected in three aspects: reducing the damage of lung tissue, promoting the transformation of macrophages toward the M2 phenotype, and promoting the transfer of mitochondria to airway epithelial cells. In addition, preclinical studies have highlighted that different MSC sources and administration routes can reduce elastase‐induced lung injury to different degrees.

In recent years, clinical trials have shown that the combination of endobronchial valve insertion and intrabronchial stromal cells (i.e., MSCs) is relatively safe for treating critically ill patients, providing support for MSC therapy as a companion therapy.^11^ A phase I pilot clinical study showed that systemic MSC infusion may help reduce inflammation in patients with COPD.^12^ In a subsequent phase 1 and 2 clinical study, four doses of umbilical cord tissue source‐MSC treatment were shown to significantly alleviate the severity of COPD symptoms.^18^

### 3.4 Asthma

Asthma is a chronic inflammatory disease of the airways caused by endogenous (such as chronic inflammation and oxidative stress) and exogenous factors (such as exposure to CS and air pollution), involving multiple cells and cellular components.^70^ The pathological process of asthma mainly involves lung structural cells and effector cells. Effector cells can secrete inflammatory factors that cause inflammation, mediate peripheral tissue destruction, induce high secretion of airway mucus, hypertrophy of smooth muscle, and increase of goblet cell levels. Asthma mainly features airway inflammation, hyper‐responsiveness, and remodeling.^71^ Repeated damage and repair of airway and lung tissue accelerate the process of airway remodeling, which will cause continuous damage and inflammation of the airway, aggravating asthma.

In preclinical research on asthma, asthmatic rats/mouse models induced by ovalbumin are generally used. These studies have shown that therapy based on MSCs has a good effect in experimental asthma models (Table 5). In the treatment of asthma, MSCs have been shown to revert pathological changes and intratracheal inflammation.^13^ In asthmatic animals, MSCs can effectively reduce pathological changes observed in lungs, such as basement membrane epithelial thickness, subepithelial smooth muscle thickness, and increase goblet cell number, to reduce airway inflammation, hyper‐responsiveness, and remodeling, thereby improving lung function and showing therapeutic potential for use in clinical settings.^14^, ^15^, ^16^, ^17^

### 3.5 Autoimmune lung diseases

Autoimmunity is a misdirected immune response in which the immune system attacks its own body. Systemic autoimmune diseases, such as systemic lupus erythematosus, systemic sclerosis, Crohn's disease, and rheumatoid arthritis, are often complicated by autoimmune lung injury like interstitial lung disease (ILD), causing fibrosis and inflammation in the lung parenchyma.^18^, ^72^ These autoimmune diseases usually occur in genetically susceptible individuals and thus, external antigens could trigger a misdirected immune response in lungs or other organs.^73^ Supporting this, Xiao‐Jun Guan et al.^74^ observed that the presence of ILD is related to human leukocyte antigen class II HLA‐DRB1*03‐DQA1*05‐DQB1*02 but not anti‐Jo‐1 auto‐antibodies or myositis subtype for myositis patients. Although immunosuppressive and anti‐inflammatory drugs like corticosteroids and therapy for modulating cytokines, such as 1L‐1β, have been widely used, there is a lack of effective treatment for autoimmune lung diseases.

MSCs‐based therapy appears to be a potential approach to treat lung disorders. Liu et al.^75^ defined the immunopathology involved in lung exacerbation during autoimmunity and determined the role of MSCs in reversing the pathological changes associated with these disorders. They found that the addition of MSCs isolated from BM of normal individuals (HBMSCs) increased the number of regulatory T cells and concomitantly reduced the cytotoxic T cell count. Moreover, HBMSCs also decreased proinflammatory chemokine/cytokine secretion, which would help to block IPF progression. Due to the immunomodulatory and regenerative properties of MSCs, their clinical efficacy and safety has been tested in a range of autoimmune disorders. Currently, over 49 ongoing trials are evaluating the efficacy of MSCs in autoimmune diseases.^76^ Generally, no unwanted side effects are observed in MSC‐treated patients and 7 of the 11 trials have demonstrated the potent immunomodulatory effects of MSCs.^77^

### 3.6 Lung cancer

Lung cancer is a malignant tumor of lung tissues characterized by uncontrolled cell growth. Globally, 1.8 million people are diagnosed with lung cancer and 1.6 million die of the disease every year. Despite commendable progress in cancer detection and therapy, prognosis of patients with lung cancer remains poor.^78^ MSCs‐based therapy has been recognized as a promising approach to treat lung cancer. The antitumor capacity of MSCs has been proven effective in different tumors, including lung cancer.^38^, ^79^, ^80^, ^81^ Furthermore, MSCs have the ability to migrate to both primary tumors and metastatic sites, which helps them exert effective tumor‐suppressing effects.^82^, ^83^

MSCs have been found to have diverse effects on tumor cells. Some studies have reported contrasting effects of MSCs with pro and antitumor properties. For example, Nemeth et al.^84^ reported contradictory effects of human MSCs (hMSCs) on tumor growth. They found that hMSCs and hMSC‐conditioned media (CM) reversed the invasion and proliferation of human A549 lung adenocarcinoma and Eca‐109 esophageal cancer cells and induced apoptosis. However, they also observed that hMSCs promoted tumor formation by enhancing vessel formation. Moreover, human UC MSC‐CM can promote epithelial‐mesenchymal transition (EMT), migration, and invasion, but also inhibited proliferation and promoted apoptosis of lung cancer cells at the same time.^85^ This study showed that the contrasting effects of MSCs are mediated by exosomes. Therefore, tumor‐promoting effects can be inhibited by blocking the release of exosomes. Besides, knockdown of TGF‐β1 expression in MSCs can reverse the EMT and enhance the antiproliferative and proapoptotic effects. Collectively, these observations suggest MSCs to be a potentially safe therapy for lung cancer.^85^

The inherent property of MSCs to migrate to tumor sites makes them suitable to deliver anticancer drugs.^86^ Several studies have provided evidence that MSCs can be used to deliver antitumor drugs, such as PTX^87^ and DOX,^88^ as well as immunomodulatory factors like IL‐12,^89^ IL‐24,^90^ IFN‐β,^91^ IFN‐γ,^92^ TRAIL^91^ to target cells. Hence, modified MSCs can target tumors and accumulate in neoplastic tissues, thereby inhibiting the tumor growth and lung tumor metastasis. Based on the results of the preclinical studies, an ongoing phase I/II clinical trial is evaluating the antitumor efficacy and safety of TRAIL‐expressing MSCs with the combination of cisplatin and pemetrexed in NSCLC (ClnicalTrial.gov^76^ Identifier: NCT03298763).

Notably, administered MSCs also show bi‐directional effects on the treatment of lung cancer. Therefore, there is a need to be cautious about the prospects of MSCs‐based therapy for lung cancer. It is necessary to understand the biology of different types of MSCs and their function in various tumor environments to reduce the adverse effects while carefully harnessing their beneficial effects.

### 3.7 The H7N9 avian influenza

In the spring of 2013, a novel avian‐origin influenza A (H7N9) virus appearednd spread in China. H7N9 viruses, the product of reassortment of viruses that are of avian origin, can quickly spread between mammalian hosts^93^ and confer the risk of human‐to‐human transmission.^94^, ^95^ The novel H7N9 virus binds to host cell receptors through its surface hemagglutinin to infect cells, but the key molecular basis of virus transmission has not yet been fully elucidated.^96^ The novel H7N9 virus can cause severe illness, including acute pneumonia and ARDS, it can also cause the extrapulmonary diseases, including rhabdomyolysis and encephalopathy, through cytokine storms.^97^, ^98^

In terms of treatment, a universal influenza vaccine is not available, though several influenza types ‐, subtype‐, and strain‐specific vaccines are available and provide protection.^99^ Antiviral therapy cannot improve the survival rate of severely patients, such as ARDS patients caused by H7N9. A study has shown^65^ that MSC therapy is a safe and effective therapy for patients with severe lung diseases induced by H7N9, and can significantly improve lung injury and fibrosis caused by ARDs. MSCs have the ability to reduce inflammatory effects and defend against cytokine storm. Its good adjuvant treatment effect may be related to the significant reduction of procalcitonin, serum creatinine, and creatine kinase levels in patients. In order to understand the potential of MSCs for treating H7N9‐induced ARDS, further elucidation of the pathogenic mechanism of H7N9 is still needed.

### 3.8 The COVID‐19

SARS‐CoV‐2 virus is a new coronavirus that has a 5% genetic association with SARS and is a subset of Sarbecovirus.^100^ And the name “COVID‐19,″ given by the World Health Organization (WHO), refers to the disease (particularly the pneumonia) associated with the SARS‐CoV‐2 virus. Several studies have shown that Angiotensin‐converting enzyme 2 (ACE2) is the main host cell receptor for SARS‐CoV‐2 entry.^101^, ^102^ The cellular protease TMRRSS2 is also necessary for the entry of coronavirus into host cells, by which the virus use to prime the S protein.^103^ Many human cells, especially alveolar type 2 (AT2) and capillary epithelium, are angiotensin I converting enzyme 2 (ACE‐2) receptor‐positive cells.^103^This is why the respiratory system is the main system through which viruses infect the human body.The inflammatory response caused by SARS‐CoV‐2 infection is the main mechanism for the body to eliminate the virus, while is also the main mechanism for SARS‐CoV‐2 to cause tissue damage and dysfunction to the body and the severity of the response is proportional to the expression of ACE‐2.^104^, ^105^ The invasion of the virus will cause the secretion of a variety of inflammatory factors, including IL‐2, IL‐6, IL‐7, IL‐17, TNF‐α, and INF‐γ. Cytokine‐induced storm results in pulmonary edema, dysfunction of air‐exchange, ARDS, even acute cardiac injury, and leading to death.^106^, ^107^

Aiming at the pathogenic mechanism of SARS‐CoV‐2, MSCs possess the potential to treat COVID‐19 as a cell therapy. As mentioned, following the COVID‐19, the patient's immune system will produce an excessive immune response, causing tissue damage. And MSC therapy can revert the complex cytokine storm, which reduces the level of many inflammatory factors including TNF‐α^108^ and promotes the endogenous repair of damaged tissues through its stem cell repair properties.^26^, ^109^ Specifically, MSCs could recover the pulmonary microenvironment, protect alveolar epithelial cells, inhibit the process of pulmonary fibrosis, and cure lung dysfunction and COVID‐19 pneumonia.^110^ There have been many case reports and clinical studies confirming that MSCs are safe and effective for the treatment of COVID‐19 patients, especially acute patients, to improve their clinical symptoms and immune function levels.^108^, ^110^, ^111^ Shi Land his colleagues have performed a phase 2 trial that proved the efficacy and safety of human UC‐MSCs for severe COVID‐19 patients’ treatment.^112^ However, current research has also highlighted many urgent problems to be solved, which include but are not limited lacking unified source of MSCs, dose, and dosing strategies, such as the number and timing of administrations.

## 4 DISCUSSION

### 4.1 Strengths and weaknesses of MSCs‐based therapy

In the last 20 years, several immunomodulatory therapies have been investigated for inflammatory diseases in the respiratory system, although only a few have progressed to clinical application. Several immunomodulation‐based therapies, such as mechanical nebulization, and drug treatment have weak treatment effects combined with side effects, such as diabetes, cataracts, gastrointestinal toxicity, and growth impairment.^113^, ^114^, ^115^ MSCs‐based therapy is considered a promising treatment for acute and chronic respiratory diseases. Thus, the application of MSCs‐based therapy is expected to reduce side effects and improve therapeutic effect.^115^

MSCs‐based therapy has obvious advantages. From a therapeutic point of view, the unique advantages of MSCs‐based therapy include the homing, migration, and regenerative properties of MSCs, which allow them to be automatically recruited to the injury site and replace damaged tissue through differentiation and regeneration when systemically administering drugs to patients.^116^, ^117^ Additionally, MSCs can regulate the inflammation of lung tissues by stimulating the reduction of proinflammatory marker cytokines and increasing anti‐inflammatory ones to restore the balance of cytokine network.^118^, ^119^

In ALI, lung injury, fibrosis, and inflammatory reactions usually occur jointly. Treatment with modified MSCs can reduce the characteristic lung injury, fibrosis, and inflammation. As these injures are usually interrelated, inhibition of one pathway may affect another, which is also a strength of MSCs.^120^, ^121^, ^122^ MSCs‐based therapy has been proven effective in multiple lung injury models, such as acid injury, *E. coli* endotoxin‐induced ALI, ventilator‐induced lung injury, hypoxia‐induced lung injury, systemic sepsis, and respiratory virus infection.^120^, ^123^, ^124^, ^125^, ^126^, ^127^, ^128^ From a management point of view, owing to its inherent low immunogenicity and limited tumorgenicity risk and ease of management, MSCs can be easily isolated and expanded in culture,^129^, ^130^ promoting their clinical application.

However, the efficacy of MSCs‐based therapies varies significantly, which may be attributed to significant differences in protein profile of the lung microenvironment during different stages of lung cancer. The lung microenvironment determines the beneficial and harmful effects of MSCs. For example, when the IL‐6 and fibronectin content is high and the antioxidant capacity is low, using MSCs may lead to adverse results.^120^ Therefore, it is necessary to check the adaptability and contraindications before administration of MSCs. For chronic diseases, such as asthma, IPF, and COPD, MSCs have shown anti‐inflammatory effects and repair function in animal‐based experiments.^26^, ^131^, ^132^, ^133^ Clinical studies have also reported their safety and few adverse effects, as well as improvement of the clinical condition and quality of life in such patients.^132^, ^133^, ^134^ However, the MSC perfusion cannot significantly improve lung function and quality of life in patients with moderate to severe disease.^117^ In some diseases, owing to the low level of specific biomolecules and growth factors, MSCs exert a weaker inhibitory effect on the remodeling process, which is more apparent in asthma.^135^, ^136^ This also implies the importance of screening patients for assessing their responsiveness to MSC treatment before metastasis.^137^

However, despite the commendable advances made in the field of MSC therapy, there are several obstacles in the clinical application of MSCs related to the cell preparation, fitness, and functionality of MSCs, as these characteristics are significantly affected by their source, cell culture method, expansion level, dosage of administration, storage, and transportation conditions.^138^, ^139^ Transplanted MSCs have a low survival rate in the recipient tissue, which may be mainly affected by the culture conditions or may cause cell death owing to the lack of proper interconnection, which restricts their application.^140^ Meanwhile, as the mechanism of action of MSCs has not been elucidated and knowledge of immunobiology is not yet perfect, the results of clinical trials often cause controversy.^120^, ^137^ In addition, the long‐term benefits of MSCs are currently unclear, and results are highly dependent on differences among patients.^141^ These uncertainties hinder the clinical application of MSCs.

### 4.2 Perspectives on current research in MSC‐based therapy

#### 4.2.1 Preclinical trials

Several preclinical studies based on MSCs have demonstrated the enormous potential of MSCs as treatment for acute respiratory diseases, such as ALI, and chronic respiratory diseases, such as asthma, COPDs, and IPF.^26^, ^131^, ^133^, ^142^ In these studies, the choice of source and animal model has a significant influence on the experimental results. In addition to efficacy, the safety issue is also key to determine if the method can be clinically used on a large scale. Therefore, in this section, we will discuss the sources, animal model, and safety in preclinical trials.

#### 4.2.2 Sources

MSCs can be obtained from several sources, such as BM‐MSC, AT‐MSC, UC‐MSC, menstrual blood (MB‐MSC), and UC tissue, that is, Wharton's Jelly‐MSC, and each MSC type has its own characteristics. For instance, BM‐MSCs have excellent properties, and are currently the most widely used MSCs.^143^ However, they require invasive acquisition and have relatively limited applicability. Meanwhile, AT‐MSCs are also promising for anti‐inflammatory and regeneration purposes,^144^ and these cells are easily obtained. Importantly, AT‐MSCs can achieve auto‐transplantation.^145^ UC‐MSCs‐based therapy has no risk for ethical issues and is isolated in a noninvasive method. Regarding physiological properties, UC‐MSCs show lower immune rejection and significantly higher growth kinetics and clonal index than those of other MSCs.^146^, ^147^

As mentioned earlier, MSCs often cause controversy in clinical trial results, which have a certain degree of relationship with the MSC source. Although all MSCs have similar general characteristics, cells from different sources may show significant differences in anti‐inflammatory or regenerative potential, depending on the specific injury at a specific location.^148^ Several studies have analyzed the differences among cells from different sources regarding their mechanisms,^146^, ^149^, ^150^ but clinical data are still lacking. Therefore, there is an urgent need to evaluate the therapeutic effects of MSCs from different sources for different diseases. For example, Antunes et al.^10^ tested the effects of three MSC sources and different administration routes on lung inflammation and remodeling in experimental emphysema. They found that MSCs from different sources variably improved elastase‐induced lung damage, but only IV administration of BM‐MSCs resulted in better cardiovascular function. Additional preclinical and clinical research on MSCs from different sources will help us standardize the research procedure and treat them symptomatically.

In addition, age and health issues of MSC donors also need to be considered. For example, AT‐MSCs from young mice rather than old mice can prevent bleomycin‐induced pulmonary fibrosis in old mice.^151^

#### 4.2.3 Safety

Manoj M Lalu et al.^152^ used meta‐analysis to evaluate immediate adverse reactions, including acute injection toxicity, fever, and adverse events in multiple organ systems, to first‐generation MSC product clinical trial security, and showed acceptable safety of MSC‐based therapy. However, the mechanism of action and clinical efficacy of MSC‐based therapy is still poorly understood,^138^, ^153^, ^154^ and safety studies need to be precise at the disease level. In vitro experiments have observed genetic instability in the passage of MSCs,^155^, ^156^ which has also caused concern about the risk of malignant proliferation and differentiation of MSCs. Therefore, we need high‐resolution genetic analysis to determine the possibility of MSC transformation after long‐term cultivation, including single‐nucleotide polymorphism, copy number variation, and insertion/deletion changes. It is also necessary to consider mutations without survival and growth advantages, which may be difficult to detect.^157^ Although MSC‐based therapy is generally safe, large‐scale animal experiments and clinical experiments are still needed to tackle the safety issues of MSCs‐based therapy.

#### 4.2.4 Animal model

Our understanding of the mechanisms of action of MSCs mainly comes from animal models represented by mice. Although several peer‐reviewed scientific reports have demonstrated the adoptive transfer of MSCs in preclinical mouse disease models, MSCs impact on mouse outcomes has not yet been converted to an equivalent in human phase III clinical trials. The inconsistency between mouse experimental results and human clinical results may be explained by (1) immunocompatibility, (2) administration, and (3) significant fitness differences in cultured adapted MSCs.^138^ Whether the mouse models themselves can effectively and accurately fit the disease model also remains a problem. For example, bleomycin‐induced pulmonary fibrosis is still considered to be the best animal model available in preclinical testing for IPF.^158^, ^159^ However, the treatment given in the first 7 days after exposure to bleomycin may work mainly by preventing the inflammatory cascade rather than reversing fibrosis, thereby limiting its applicability to human IPF.^160^ In addition, current research lacks an animal model that can represent chronic IPF,^158^, ^161^ which adds additional obstacles to IPF research. Moreover, the mice modeling method brings uncertainty to the experimental results. For example, lung injury has several models, such as the acid injury model, *E. coli* endotoxin‐induced, ventilator‐induced, and hypoxia‐induced lung injury model, systemic sepsis model, and respiratory virus infection model.^120^, ^123^, ^124^, ^125^, ^126^, ^127^, ^162^ The performance of MSCs‐based therapies differs among these models, indicating that a single animal model cannot be used to represent a class of diseases. Thus, animal models need to be further standardized to the disease, with as much detail as possible, that is, we need more accurate and standardized mouse models to help us build acute and chronic disease models with different stages and different pathogenic factors, and tightly integrate animal models with clinical experiments to improve preclinical studies.

In the past decade, the exploration of MSC‐based therapies has gradually evolved into a standardized development, and an increasing number of preclinical research results have advanced to clinical research. Therefore, it is particularly critical to reassess the safety and effectiveness of preclinical studies of MSCs‐based therapy through regulatory means,^153^, ^163^ considering details, such as cell expansion conditions, medium composition, cell treatment protocols, administration dosage, MSC source, and animal model.

#### 4.2.5 Clinical trials

As MSCs‐based therapies have made considerable breakthroughs in preclinical research, several of them have progressed to clinical studies with more than 800 registered clinical studies being conducted on MSCs‐based treatment approaches worldwide,^153^ according to Clinical Trails Data Bank at the National Institutes of Health clinical trials (Table 6).

**TABLE 6:** The clinic trials of inflammatory diseases in the respiratory system over last decade

Diseases	Time	Cell source	Study	Route	Status	Outcome	NCT number
IPF	2013	PD‐ MSC	A Study to Evaluate the Potential Role of Mesenchymal Stem Cells in the Treatment of Idiopathic Pulmonary Fibrosis (MSC in IPF)	i.v.	Completed	No severe negative effect	NCT01385644
	2014	AT‐MSC	Evaluate Safety and Efficacy of Intravenous Autologous AT‐MSCs for Treatment of Idiopathic Pulmonary Fibrosis	i.v.	UN	NYR	NCT02135380
	2017	BM‐MSC	Role of Stem Cell Therapy in Interstitial Pulmonary Fibrosis	i.v.	Completed	NYR	NCT03187431
	2018	BM‐MSC	Safety and Efficacy of Allogeneic Mesenchymal Stem Cells in Patients With Rapidly Progressive Interstitial Lung Disease	i.v.	NYR	NYR	NCT02594839
	2018	BM‐MSC	Study of Autologous Mesenchymal Stem Cells to Treat Idiopathic Pulmonary Fibrosis (CMM/FPI)	v.FOB	UN	NYR	NCT01919827
	2019	UC‐MSC	A Study on Radiation‐induced Pulmonary Fibrosis Treated With Clinical Grade Umbilical Cord Mesenchymal Stem Cells	v.FOB	Completed	NYR	NCT02277145
ALI	2014	AT‐MSC	Adipose‐derived Mesenchymal Stem Cells in Acute Respiratory Distress Syndrome	i.v.	UN	NYR	NCT01902082
	2014	Mens‐MSC	Using Human Menstrual Blood Cells to Treat Acute Lung Injury Caused by H7N9 Bird Flu Virus Infection	i.v.	ongoing	NYR	NCT02095444
	2014	BM‐MSC	Treatment of Severe Acute Respiratory Distress Syndrome With Allogeneic Bone Marrow‐derived Mesenchymal Stromal Cells	NR	UN	NYR	NCT02215811
	2015	UC‐MSC	Human Umbilical‐Cord‐Derived Mesenchymal Stem Cell Therapy in Acute Lung Injury (UCMSC‐ALI)	i.v.	UN	NYR	NCT02444455
	2015	BM‐MSC	Human Mesenchymal Stem Cells For Acute Respiratory Distress Syndrome (START)	i.v.	completed	Ref	NCT01775774
	2016	BM‐MSC	Mesenchymal Stem Cell in Patients With Acute Severe Respiratory Failure (STELLAR)	i.v.	UN	NYR	NCT02112500
	2019	BM‐MSC	Human Mesenchymal Stromal Cells For Acute Respiratory Distress Syndrome (START) (START)	i.v.	completed	Ref	NCT02097641
	2019	Multiple stem cells	A Phase 1/2 Study to Assess MultiStem® Therapy in Acute Respiratory Distress Syndrome (MUST‐ARDS)	NR	completed	NYR	NCT02611609
	2019	UC‐MSC	Mesenchymal Stem Cells for Multiple Organ Dysfuntion Syndrome After Surgical Repair of Acute Type A Aortic Dissection	i.v.	recruiting	NYR	NCT03552848
	2019	UC‐MSC	Human Umbilical Cord Mesenchymal Stem Cells (MSCs) Therapy in ARDS (ARDS)	i.v.	recruiting	NYR	NCT03608592
	2020	BM‐MSC	Mesenchymal Stromal Cells For Acute Respiratory Distress Syndrome (STAT)	i.v.	recruiting	NYR	NCT03818854
	2020	BM‐MSC	Mesenchymal Stem Cells (MSCs) for Treatment of Acute Respiratory Distress Syndrome (ARD) in Patients With Malignancies	i.v.	completed	NYR	NCT02804945
	2020	UC‐MSC	Repair of Acute Respiratory Distress Syndrome by Stromal Cell Administration (REALIST) (COVID‐19) (REALIST)	i.v.	recruiting	NYR	NCT03042143
Asthma	2020	hMSC	Allogeneic Human Cells (hMSC) Via Intravenous Delivery in Patients With Mild Asthma	i.v.	Active, not recruiting	NYR	NCT03137199
	2018	UC‐MSC	Safety and Feasibility Study of Intranasal Mesenchymal Trophic Factor (MTF) for Treatment of Asthma	i.v.	Active, not recruiting	NYR	NCT02192736
COPD	2020	MSC	Mesenchymal Stem Cells in the Treatment of Subjects With Advance Chronic Obstructive Pulmonary Disease (COPD)	i.v.	Recruiting	NYR	NCT04047810
	2020	UC‐MSC	Mesenchymal Stem Cells for The Treatment of Chronic Obstructive Pulmonary Disease	i.v.	Active, not recruiting	NYR	NCT04047810
	2020	BM‐MSC (PROCHYMAL)	PROCHYMAL™ (Human Adult Stem Cells) for the Treatment of Moderate to Severe Chronic Obstructive Pulmonary Disease (COPD)	i.v.	Completed	no infusional toxicities, no deaths or severity, no significant adverse effect, no significant differences in PFTs or quality‐of‐life indicators; CRP decrease in early stage	NCT00683722
	2020	ATSC	Safety, Tolerability and Preliminary Efficacy of Adipose Derive Stem Cells for Patients With COPD	i.v.	Terminated	NYR	NCT02161744
	2019	BM‐MSC	Cell Therapy Associated With Endobronchial Valve	i.v.	Not yet recruiting	NYR	NCT04018729
	2017	ATSC	Safety and Efficacy of Adipose Derived Stem Cells for Chronic Obstructive Pulmonary Disease	i.v.	Completed	NYR	NCT02216630
	2016	AT‐MSC (hADAS)	Adipose Derived Stem Cells Transplantation for Chronic Obstructive Pulmonary Disease	i.v.	UN	NYR	NCT02645305

i.v.: Intravenous; NR: not reported; UN: Unknown; NYR: Not yet reported; NCT number: The national clinical trial number; AT‐MSC: Adipose tissue derived mesenchymal stem cells; BM‐MSC: Bone marrow mesenchymal stromal cells; UC‐MSC: Umbilical cord mesenchymal stem cells; PD‐MSC: Placenta‐derived MSC.

Owing to the difference between the animal model and the actual clinical scenario,^138^ while conducting clinical research based on preclinical studies, regulatory issues, such as cell source, dosage, and disease stage, should be considered, since these factors can directly lead to different results under the same therapy regimen. For example, a large part of IPF research has explored the therapeutic effects of MSC‐based therapy during the early stages of inflammation rather than the late stage of fibrosis, which implies that MSC administration only prevents IPF and cannot be used as a late‐stage treatment.^164^ Given the limitations of current clinical research regarding the sample size and variable control, more high‐quality clinical research studies are warranted that are based on large samples, are randomized and double‐blinded, and control variables to help clinical frontline workers understand MSC‐based therapy and improve its efficacy in the future. Moreover, we need to classify and stage the disease in patients, so that they can receive targeted and precise treatment. Unfortunately, MSCs‐based therapy is not devoid of ethical issues, and double‐blinded clinical research may be difficult. However, we advocate the introduction of optimized procedures to standardize the variables in MSCs‐based therapies, such as cell culture conditions and MSC sources.

### 4.3 Improvement of MSCs‐based therapy

#### 4.3.1 Combination therapy

To maximize the therapeutic effect, the combination of MSC and traditional drug therapy to produce a synergistic effect is a frequently used solution in clinical practice. For example, for IPF treatment, the two pharmacological targets approved by the US Food and Drug Administration are TGF‐β (pirfenidone) and PDGF/VEGF/FGF (nintedanib). The efficacy of pirfenidone is impressive. Compared with those in the placebo group, pirfenidone can significantly reduce inflammation and prolong survival, but cannot reproduce the normal structure of lung tissue.^165^ MSCs‐based therapy has the function of tissue repair, which can make up for the defects of targeted drug therapy to a certain extent. In addition, because the pathogenesis of these lung diseases, especially chronic diseases, involves multiple coactivation pathways, targeted therapy is unlikely to be effective in isolation.^166^ Combining traditional and MSCs‐based therapy may improve therapy efficacy.^167^, ^168^ However, there are still insufficient clinical data to support the effectiveness of this therapy.

#### 4.3.2 Improve the effectiveness of MSCs

Modifying MSC homing, migration, and regenerative properties through biochemical means, gene‐editing combined with computational modeling, or with material science can improve MSCs‐based therapy efficiency. In clinical applications, MSCs may have different administration modes: systemic administration (IV or intra‐arterial [IA] injection) or local administration (intracoronary [IC] injection or direct injection to the target tissue). Among these, IV injection is the most widely used because it has a minimally invasive effect, is easy to repeat infusion, and the cells will remain close to oxygen‐rich and nutrient‐rich blood vessels after infiltrating the target tissue.^169^ Although MSCs have homing ability, this process does not seem to be very efficient. Therefore, it is vital to deliver MSCs to target tissues accurately. A large number of preclinical studies have shown that the transplantation rate is very low, close to 5%,^117^ which is attributed to the low expression level of homing molecules, loss of expression of such molecules during amplification, and MSC heterogeneity.^170^ Several research teams are trying to improve the homing efficiency of MSCs, including changing the method of administration, changing MSC culture conditions to optimize the expression of homing molecules, modifying cell surface receptors, and modifying target tissues to enhance MSC attraction. Regarding administration method, Sakar D et al.^169^ combined MSC administration with the use of heparin, significantly reducing the capture of MSCs in the lung and increasing the capture rate of targeted sites. Considering culture methods, as MSCs downregulate the expression of homing molecules during amplification, adding cytokines to the amplification system can increase CXCR4 expression on the membrane, thereby upregulating the expression of homing molecules. These cytokines include, but are not limited to, flt3 ligand, stem cell factor, hepatocyte growth factor, IGF‐1, TNF‐α, IL‐1β, and IFNγ.^171^, ^172^, ^173^, ^174^, ^175^ Additionally, hypoxic conditions can also increase the expression of CXCR4.^176^

To improve homing efficiency, as MSCs are heterogeneous cell populations, a single‐cell sequencing method can be used to find specific subsets with a robust homing ability for separation. Cell surface engineering, that is, the transient modification of the cell surface, also improves the homing ability of MSCs,^177^ and several research groups have developed different techniques for MSC surface modification.^178^, ^179^, ^180^ In addition, the target tissue can be modified by chemical, magnetic field, or radiotherapy to improve the homing efficiency of MSCs.^181^, ^182^

Regarding the migration and localization of MSCs in vivo, current research is mainly based on experimental measurements and clinical observations. These experiments attempt to elucidate the processes involved in migration from statistical results, including cell signaling and protein interactions.^183^ However, this process is complicated, mainly owing to the low frequency of stem cells and the lack of quantitative tracking experimental techniques for stem cell migration in vivo. Different mechanical/physical stimuli (such as shear flow, compressive force, stiffness, tension, and geometry) and various biochemical factors (such as the concentration and distribution of chemokines) are difficult to study through controlled variables using experimental methods.

In recent years, computational modeling has become a compelling method, making up for the lack of experimental methods, to understand the migration process of stem cells and help them accurately migrate to target tissues. When applying computational modeling to MSC research, the model should address the differences in migration kinetics between stem cells and other cells, and those between MSCs and other stem cells, considering whether they differentiate or not and the diversity of stem cells, respectively. Simulations of MSC migration kinetics in the local microenvironment in vitro^184^, ^185^ showed how numerical simulation can help us obtain unknown cell behaviors and tissue functions of small samples, study the process of MSCs to target damage sites, and design mechanisms to increase the number of cells targeted to damaged tissues.^186^ While the migration process of a single cell is relatively easy to understand, in a real situation, the migration of multiple cells should be considered. Understanding the protein expression and signaling network in migrating cells will help to model and analyze collective migration.

In general, the development of computational modeling is still insufficient, mainly owing to data scarcity and uncertainty about the MSC mechanism caused by the lack of physical and biochemical sensor technologies and basic research, respectively. Stem cell models are roughly divided into deterministic models, stochastic models, and hybrid models. Deterministic models are mainly based on the concept that stem cells respond to differentiation stimuli in a dose‐dependent manner and therefore have deterministic behaviors. Contrastingly, stochastic methods use probability functions to describe events, such as differentiation and diffusion.^187^ Therefore, understanding the MSC mechanism can help improve deterministic models, and data collection can help improve random and hybrid models. Meanwhile, the explosion of data and the improvement of computing power can also bring about essential changes in these models based on the concept of big data. We believe that in the near future, with the continuous collection of MSC in vivo data and increased understanding of MSC‐related mechanisms, computational modeling will be significantly strengthened and improved to assist us enhancing MSC migration activities.

In addition to strengthening the homing and migration functions of MSCs, it is also necessary to regulate their differentiation and regeneration ability and immunological characteristics. Under normal culture conditions, MSCs usually proliferate through cell division, unless induced by special differentiation conditions. Moreover, through high‐density inoculation of MSCs, the expression of some genes related to immunosuppressive properties can be increased.^188^, ^189^ Combined with the rapid development of gene‐editing technology in recent years, we can now easily genetically modify MSCs. Using CRISPR‐Cas9 technology,^190^, ^191^ additional available MSC editing technologies have been developed. For example, by combining nanotechnology, we can design an exosome‐liposome hybrid nanoparticle to easily provide the CRISPR/Cas9 system in MSCs.^192^ Furthermore, with the development of gene therapy, the combination of genetic modification and MSC‐based therapy has become a trend. For example, ALI can be treated by UC‐MSCs with a modified Angiopoietin‐1 gene.^193^ Besides, the combination of MSC‐based therapy and tissue engineering technology can also enhance MSC repair and regeneration functions. For example, Yang et al. combined MSCs with allogeneic cartilage tissue engineering to discuss the proliferation, differentiation, and secretion of various soluble factor functions of MSCs in a three‐dimensional environment to inspire scaffold design and achieve the required tissue regeneration.^194^ Moreover, gene‐editing technology can also be used to adapt the existing two‐dimensional environment‐adapted MSCs to the three‐dimensional environment, promote tissue generation, and reduce immune rejection.^195^ Additionally, modeling the lungs of patients and implement in vitro 3D‐tissue culture is a promising strategy for some diseases with severe lung necrosis that require transplantation, such as advanced IPF. Hence, it is necessary to establish a highly integrated 3D network of different lung cell types, including mesenchymal, epithelial, fibroblast, endothelial, inflammatory, and neuronal cells.

#### 4.3.3 Cell‐free therapy

In clinical trials, MSCs‐based therapies usually display a low migration rate and low survival rate of MSCs in the recipient tissues.^140^ However, this does not seem to have a significant impact on MSC function, which supports the hypothesis that their secretions may mediate the regenerative capacity. As mentioned above, MSC paracrine effects have been widely studied; their secretions are mainly responsible for the interaction between MSCs and target cells.^196^, ^197^ On this basis, research on MSC‐CM and EV has gradually received attention. Several studies have used different animal models to prove that CM can mimic the anti‐inflammatory mechanism of parental MSCs. For example, in acute and chronic asthma, intranasal infusion of BM‐MSC‐CM can reduce the levels of inflammatory cytokines IL‐4 and IL‐13 in the lungs and increase the level of IL‐10.^198^ Shen and colleagues studied the protective effect of BM‐MSC‐CM on bleomycin‐induced lung injury and fibrosis in vitro (A549 alveolar epithelial cells) and in vivo (rat model). After treatment with MSC‐CM, A549 cells were significantly protected from bleomycin‐induced apoptosis, while lung inflammation, fibrosis scores, collagen deposition, and apoptosis in rats challenged with bleomycin were reduced.^199^ In a rat model of COPD, MSC‐CM administration can reduce emphysema caused by CS and increase the number of small pulmonary vessels and MSCs.^200^ However, the main limitation of MSCs‐CM is the relatively rapid degradation of biologically active molecules. Additionally, as MSC‐based therapies, it lacks standard procedures for regulations.^201^

EVs provide another popular cell‐free therapy. They can stably transport biologically active substances without being hydrolyzed or mutated, and several teams have demonstrated the anti‐inflammatory effects of EVs in ALI,^26^ asthma,^202^ IPF,^28^, ^203^ and other diseases. In the neonatal rat model, MSC‐derived EVs are as effective as parental MSCs, which can confer VEGF‐mediated protection against activated macrophages, proinflammatory cytokines, and increased cell death.^204^ In the neonatal rat model, EVs derived from MSCs are as effective as parental MSCs. This protective effect is mainly mediated by the transfer of VEGF contained within EVs.^204^ Recent evidence shows that EVs derived from MSCs can carry miRNAs with antiapoptotic properties and can promote the reduction of lung inflammation,^205^, ^206^ thus being a promising method to reduce inflammation. Compared with traditional MSCs‐based therapies, EVs are more stable, easily enter the systemic circulation, and maintain long‐lasting high concentrations.^207^ Moreover, cell‐free therapy also reduces the risk of tumor formation and has advantages similar to those of traditional medicine in terms of transportation, storage, and standardization. However, EVs have a high degree of heterogeneity, and the content of stored bioactive compounds varies greatly, which adds to the challenge of their dosage. In general, cell‐free therapy is safer, and has advantages over cell therapies in variable control and standardization, with a strong potential for immunomodulation and anti‐inflammatory effects in lung diseases.

## 5 CONCLUSION

All in all, the effectiveness of MSC‐based therapy for respiratory diseases has been proven by preclinical studies and clinical trials. However, limitations of related research need to be worked out to explore their clinical applications. In conclusion, MSC‐based therapy has provided a promising strategy for the treatment of respiratory diseases.

## CONFLICTS OF INTERESTS

The authors have declared that no competing interest exists.

## AUTHOR'S CONTRIBUTIONS

MY Wang, TY Zhou, and ZD Zhang contributed equally to the review, and should be viewed as cofirst authors, they drafted and revised the manuscript, and did the bulk of literature search and categorization of references. HY Liu and ZY Zheng contributed by summarizing related researches guiding the framework, placing the points in context, and constantly giving invaluable feedback. HQ Xie provided assistance and guidance during manuscript writing. All authors read and approved the final manuscript.

## ETHICS STATEMENT

Not applicable.

## Data Availability

Not applicable.
